# Evaluation of Hepatoprotective Effect of Curcumin on Liver Cirrhosis Using a Combination of Biochemical Analysis and Magnetic Resonance-Based Electrical Conductivity Imaging

**DOI:** 10.1155/2018/5491797

**Published:** 2018-05-17

**Authors:** Eun Jung Kyung, Hyun Bum Kim, Eun Sang Hwang, Seok Lee, Bup Kyung Choi, Jin Woong Kim, Hyung Joong Kim, Sang Moo Lim, Oh In Kwon, Eung Je Woo

**Affiliations:** ^1^College of Medicine, Chung-Ang University, Seoul 06974, Republic of Korea; ^2^Department of East-West Medical Science, Kyung Hee University, Yongin 17104, Republic of Korea; ^3^Impedance Imaging Research Center (IIRC), Kyung Hee University, Seoul 02447, Republic of Korea; ^4^Department of Radiology, Chonnam National University Medical School, Gwangju 61469, Republic of Korea; ^5^Department of Nuclear Medicine, Korea Institute of Radiological and Medical Sciences, Seoul 01812, Republic of Korea; ^6^Department of Mathematics, Konkuk University, Seoul 05029, Republic of Korea

## Abstract

In oriental medicine, curcumin is used to treat inflammatory diseases, and its anti-inflammatory effect has been reported in recent research. In this feasibility study, the hepatoprotective effect of curcumin was investigated using a rat liver cirrhosis model, which was induced with dimethylnitrosamine (DMN). Together with biochemical analysis, we used a magnetic resonance-based electrical conductivity imaging method to evaluate tissue conditions associated with a protective effect. The effects of curcumin treatment and lactulose treatment on liver cirrhosis were compared. Electrical conductivity images indicated that liver tissues damaged by DMN showed decreased conductivity compared with normal liver tissues. In contrast, cirrhotic liver tissues treated with curcumin or lactulose showed increased conductivity than tissues in the DMN-only group. Specifically, conductivity of cirrhotic liver after curcumin treatment was similar to that of normal liver tissues. Histological staining and immunohistochemical examination showed significant levels of attenuated fibrosis and decreased inflammatory response after both curcumin and lactulose treatments compared with damaged liver tissues by DMN. The conductivity imaging and biochemical examination results indicate that curcumin's anti-inflammatory effect can prevent the progression of irreversible liver dysfunction.

## 1. Introduction

Cirrhosis is histologically defined as an advanced fibrosis of liver tissues. Liver fibrosis is a hepatic response to chronic injury and is characterized by excess deposition of collagen, proteoglycans, and other macromolecules in the extracellular matrix [1]. These result in tissue degeneration and finally lead to portal hypertension and end-stage liver disease [1]. Histological changes from cirrhosis were considered irreversible, but recent studies have reported that liver fibrosis, even the more advanced stages, can be ameliorated with appropriate therapy [2]. The amelioration of liver fibrosis not only prevents the development of liver cirrhosis but can also reduce the incidence of hepatocellular carcinoma. Therefore, the appropriate therapy may help to extend the life span of patients with liver fibrosis from a long-term perspective.

Herbal medicine has received attention as a potential treatment for liver cirrhosis [3]. For example, extracts from *Artemisia capillaris* Thunb. reduced the production of fibrogenic factors, and a specific fragment inhibited fibroblast proliferation [4, 5]. The *Anemarrhena* rhizome has been used for the treatment of inflammatory diseases such as lung disease, fever, and diabetes. Curcumin is a yellow pigment found in the rhizome of the spice turmeric (*Curcuma longa* Linn., a member of the Zingiberaceae family). Curcumin has antioxidant, anti-inflammatory, and anticarcinogenic pharmacological effects [6]. It acts by either interacting with molecular targets directly or altering gene expression and signaling pathways. Recent studies have reported that curcumin effectively inhibits liver cirrhosis through its action on many pathways; for example, it inhibits the NF-*κ*B pathway and reduces oxidative stress [7]. Thus, curcumin has potential as a therapy for liver diseases.

Imaging techniques are widely used to objectively visualize therapeutic effects, but there still exists demand for novel contrast information prior to morphological changes. Recent developments in magnetic resonance- (MR-) based tissue property mapping techniques have improved noninvasive imaging of electrical conductivity distribution inside living tissues [8]. Magnetic resonance electrical impedance tomography (MREIT) is a bioimaging modality that can provide high-resolution conductivity images based on the current-injection MR method [8, 9]. It is well known that the electrical conductivity of biological tissues is primarily determined by the concentration and mobility of ions which existed in the intra- and extracellular structures [10]. Electrical conductivity changes are closely related to the physiological and pathological conditions of tissues or organs [10–12]. Liver tissues mostly consist of hepatocytes, which make up 70~85% of the liver mass; therefore, the variations of ion concentration and mobility inside the liver may show similar patterns [10–12]. This suggests that liver tissues can exhibit uniform distribution of electrical tissue conductivity. Therefore, MREIT is a clinically useful method to image these features.

The purpose of this study was to evaluate the effects of curcumin on liver cirrhosis using an *in vivo* rat model. After induction of liver cirrhosis, we performed MR-based electrical conductivity imaging to visualize the tissue response after different treatments. The pathological state of the liver was evaluated using *α*-smooth muscle actin (*α*-SMA) and cyclooxygenase-2 (COX-2) expression levels, and a biochemical marker of inflammation was used to estimate the functional state of liver tissues. The effects of curcumin treatment were compared with those of lactulose which is typically used as a positive effector on liver cirrhosis.

## 2. Materials and Methods

### 2.1. Animal Preparation and Drug Administration

A total of 32 Sprague-Dawley rats (8 weeks old, weighing 260~280 g) were used. The rats were fed on a standard pellet diet supplied by Orient Bio Inc. (Gyeonggi, South Korea) and housed two per cage at animal facilities. The temperature was maintained at 25 ± 1°C, and relative humidity was 60 ± 5%. The rats were subject to a 12-hour light/dark cycle (lights on 8 a.m. and off 8 p.m.). All the rats were allowed to adapt to the laboratory conditions for 1 week before the experiments. During the experiments, they were given tap water and a standard diet ad libitum and weighed each week to evaluate their state of health. The rats were divided into the following four groups: normal control: intraperitoneal (IP) injection of phosphate-buffered saline (PBS) and oral administration of distilled water (DW); DMN-only: IP injection of *N*-nitrosodimethylamine (DMN, 48670, Sigma-Aldrich, St. Louis, MO, USA) and oral administration of DW; DMN with Lac: IP injection of DMN and oral administration of lactulose; and DMN with Cur: IP injection of DMN and oral administration of curcumin.

**Figure 1:**
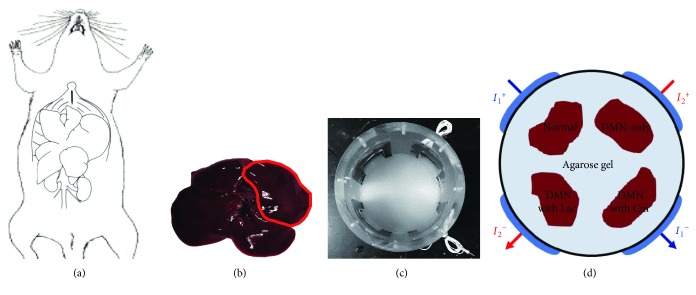
Experimental setup for MREIT conductivity imaging of liver phantom. (a) The whole liver was removed, and (b) the liver block was resected for phantom imaging. (c) A cylindrical acrylic phantom was filled with agarose gel of 1.0 S/m conductivity to support the liver block. (d) Electrodes were attached to the sides of the phantom to inject imaging currents.

To induce liver cirrhosis, a dose of 10 *μ*g/kg DMN was intraperitoneally injected twice a week for 4 weeks (dissolved in PBS). In the normal control group, PBS alone was injected in the same manner. All hepatoprotective oral treatments were conducted on the same day and for the same duration as the DMN and PBS injections referred to above. In the normal control and DMN-only groups (which received IP injections of PBS and DMN, resp.), DW was orally administered. Curcumin (C1386, Sigma-Aldrich, St. Louis, MO, USA; 100 mg/kg) and lactulose (644913501, Joong-Wae Pharma Co., Seocho, Seoul, Korea; 250 mg/kg) were orally administered to the groups that received IP injections of DMN. Curcumin was dissolved in 1% dimethyl sulfoxide at a concentration of 10 mg/mL; lactulose was not diluted [13–16]. On the 6th week after administration, animals were sacrificed and their liver blocks were resected for the MREIT imaging experiments and for biochemical evaluations, such as tests of inflammatory indicator levels and staining data (Figures 1(a) and 1(b)). All animal procedures complied with the Institutional Care and Use Committee (KHUASP(SE)-15-024) of Kyung Hee University.

### 2.2. Phantom Preparation and Imaging Experiment

A cylindrical acrylic phantom 13 cm in diameter and 16 cm in height was used for the MREIT imaging experiment. The resected liver blocks containing the fibrotic lesion (Figure 1(b)) were positioned inside the center of the phantom. The phantom was filled with conductive material (agarose gel of 0.01 S/m conductivity) to support the position of the liver block (Figure 1(c)). Four electrodes were attached to the sides of the phantom and imaging currents *I*_1_ and *I*_2_ were sequentially injected with two different directions through two pairs of opposing electrodes (Figure 1(d)). Following the phantom preparation time of over 30 minutes, the phantom was placed inside the bore of a 3T MRI scanner (Magnetom Trio A Tim, Siemens Medical Solutions, Erlangen, Germany). By this time, the phantom and liver blocks had reached thermal equilibrium at room temperature.

Using a constant current source, the first current *I*_1_ was injected between one opposing pair of electrodes. The injected current was a 3 mA of amplitude and a total pulse width of 81 msec. After acquiring the first magnetic flux density (*B_z_*) data set for *I*_1_, the second injection current *I*_2_ with the same amplitude and pulse width was injected through the other pair of opposing electrodes to obtain the second data set. A spin-echo-based multiecho pulse sequence was used to obtain the MR magnitude and magnetic flux density (*B_z_*) images [8, 9]. The imaging parameters for obtaining the conductivity images were as follows: repetition time/echo time = 1000/10, 20, 30, and 40 msec (four echoes), field of view = 160 × 160 mm^2^, slice thickness = 3 mm, number of excitations = 6, matrix size = 128 × 128, and number of slices = 8. The total imaging time to obtain the magnetic flux densities (*B*_z,1_ and *B*_z,2_) by *I*_1_ and *I*_2_ was 25 min. To reconstruct electrical tissue conductivity images, a single-step harmonic *B_z_* algorithm implemented in the software package CoReHA (conductivity reconstructor using harmonic algorithms) was used [8, 9]. After conductivity image reconstruction, we measured the conductivities of whole liver blocks in the four different groups and analyzed the values.

### 2.3. H&E Staining and Immunohistochemistry

Rats were anesthetized and transcardially perfusion-fixed with 4% formaldehyde in 0.1 M sodium phosphate buffer (pH 7.4). Liver tissues were removed quickly and postfixed with the same fixation solution overnight at 4°C. 20 *μ*m thick coronal sections of liver tissues were made using a freezing microtome (Leica, 2800N, Germany). The sections were stained with hematoxylin and eosin (H&E) for tissue morphology observation. For immunohistochemical staining of *α*-smooth muscle actin (*α*-SMA) and cyclooxygenase-2 (COX-2), free-floating liver sections were stained using 3,3′-diaminobenzidine (DAB) reaction. The sections were rinsed with 0.05 M PBS and incubated for 15 min in 1% hydrogen peroxide PBS at room temperature. The sections were incubated overnight at 4°C with primary antibody against *α*-SMA (ab7817, Abcam, Cambridge, MA, USA, 1 : 2000 dilution) and COX-2 (sc1747, Santa Cruz Biotech, Santa Cruz, CA, USA, 1 : 1000 dilution), then incubated with biotinylated anti-goat (sc2020, Santa Cruz Biotech, Santa Cruz, CA, USA, 1 : 200 dilution) and anti-mouse (sc2005, Santa Cruz Biotech, Santa Cruz, CA, USA, 1 : 200 dilution) secondary antibodies for 2 hours at room temperature, after which the avidin-biotin complex (Vector Laboratories, Burlingame, CA, USA) method was carried out with peroxidase coupling in a mixture containing 0.05% DAB (Sigma-Aldrich, St. Louis, MO, USA) and 0.03% H_2_O_2_ for 2–5 min. Images of the DAB-colorized brain sections were captured using a light microscope (BX51, Olympus, Tokyo, Japan) equipped with a CCD camera (DP70, Olympus).

### 2.4. Serum Biochemical Analysis

Blood was collected from the abdominal aorta on the final day of the experiment. After centrifuging at 3000 ×g for 20 min, the serum was separated and stored at −70°C. The serum levels of albumin (ALB), aspartate transaminase (AST), and alanine transaminase (ALT) were measured using an enzyme-linked immunosorbent assay (ELISA) kit. Samples and standards were analyzed using an auto chemistry analyzer (AU400, Olympus, Tokyo, Japan).

### 2.5. Statistical Analysis

The relative optical densities of various immunolabeled cells were measured and analyzed using ImageJ software (Ver. 1.44p, NIH, Bethesda, MD, USA). The relative optical densities were expressed by the mean gray values on an inverted black-white binary image and normalized against the values of the normal group with an area of 10^5^ *μ*m^2^. The mean values of four sections (obtained from the four groups: normal, DMN-only, DMN with Lac, and DMN with Cur) were statistically analyzed. The western blot results were converted to the percentage rate compared with the normal control, designated as 100%. The converted values were used in the statistical analysis. We used a *t*-test to compare the difference between the two groups, using SPSS 20.0 for Windows (SPSS Inc., Chicago, IL, USA). The differences were considered statistically valid when the *p* < 0.05.

## 3. Results

### 3.1. Body Weight Measurement

As the body weight change is one factor used to evaluate cirrhosis, we directly measured the body weight of all rats and compared the weight among the experimental groups (Table 1). During the experiments, body weight continually increased and was similar in all experimental groups over time, except the DMN-only group. The fluctuation in the DMN-only group may reflect tissue condition related to the degree of cirrhosis.

### 3.2. Serum Biochemical Assay Measurement

For serum biochemical analysis, we measured AST, ALT, and ALB levels, all of which are well-known indicators of liver functionality (Table 2). Comparison of normal and DMN-only groups showed that the AST and ALT levels in the DMN-only group were significantly higher than those in the normal control group, indicating the progression of liver injury. Comparison of treatment groups and the DMN-only group showed that the DMN with curcumin group exhibited significantly lower AST and ALT values than the DMN-only group, whereas the DMN with lactulose group exhibited significantly lower values only for ALT. There were no significant differences in ALB level among the experimental groups.

### 3.3. Electrical Conductivity of Liver Tissues

**Figure 2:**
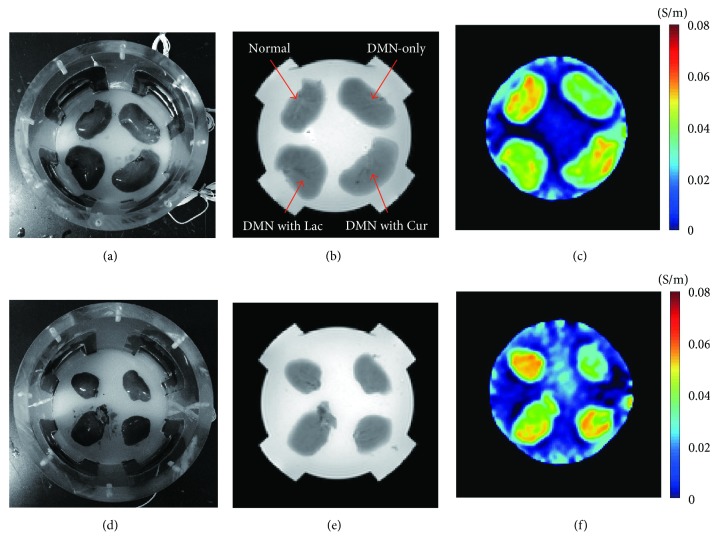
Typical MREIT images of two liver phantoms. (a and d) Phantom consisted of four liver blocks including normal control, DMN-only, DMN with lactulose, and DMN with curcumin. (b and e) MR magnitude images provide morphological information on the liver phantom. (c and f) Electrical conductivity images of liver tissue indicate contrast information depending on their tissue condition.

In order to visualize the hepatoprotective effect, we performed a MREIT conductivity imaging experiment using two liver tissue phantoms (Figure 2). Morphological information was obtained from the MR magnitude images (Figures 2(b) and 2(e)). There was no significant difference in contrast among the experimental groups. However, the conductivity images (Figure 2(c) and 2(f)) showed that the contrast was highest in normal liver tissue and lowest in DMN-only tissue. The conductivity contrast in both the treatment groups was clearly higher than that in the DMN-only tissue. Specifically, the conductivity of DMN with curcumin was slightly higher than that of DMN with lactulose. The results from both phantoms showed a similar pattern.

**Figure 3:**
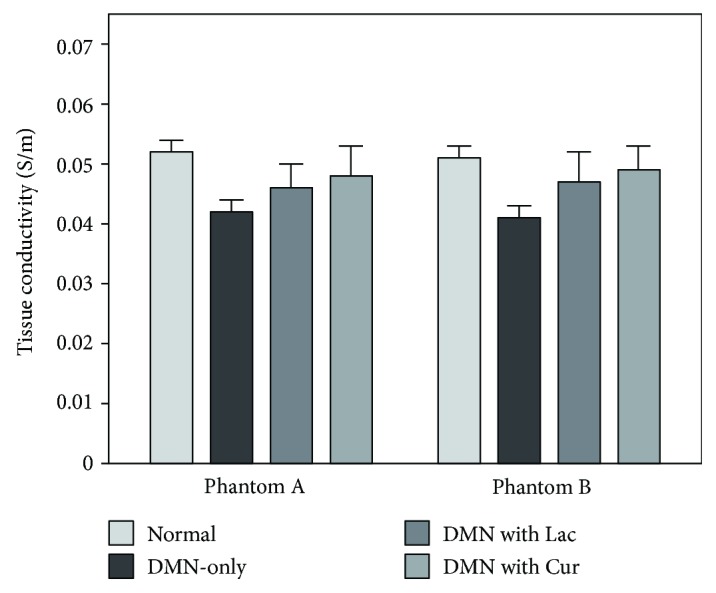
Bar graph showing quantitative analysis of conductivity contrast at four different experimental groups.

For quantitative analysis, we measured the conductivity values of all liver tissues across experimental groups (Figure 3). The conductivity values were highest in the normal control and lower in the order of DMN with curcumin, DMN with lactulose, and DMN-only groups. The conductivity in the treatment groups was significantly higher than that in the DMN-only group. Therefore, it is possible to explain that curcumin has the hepatoprotective effects from the bioelectromagnetic standpoint.

### 3.4. Histopathological Findings

**Figure 4:**
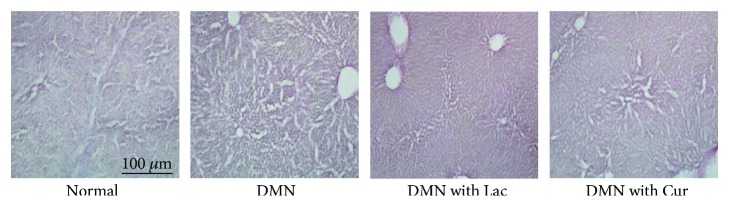
Histological observation of fibrotic liver tissues after curcumin and lactulose treatment. Sections obtained by frozen section were subjected to H&E staining. DMN (10 mg/kg) or sterile saline was injected into the rat peritoneum three times per week for 4 weeks. A solution of curcumin, lactulose, or normal saline was given daily through oral administration for 28 days.

Liver tissues from each experimental group were subjected to H&E staining and their histological morphologies compared (Figure 4). The DMN-only group tissues exhibited histological abnormalities, such as congestion and destruction of hepatic architecture, massive and severe hepatocyte necrosis, and remarked mononuclear cell infiltration. In contrast, the severity of histological abnormalities and cirrhotic liver alterations in the lactulose and curcumin treatment groups was significantly lower than that in the DMN-only group. The size and number of abnormalities in the cirrhotic liver were lower in the curcumin treatment group than in the lactulose treatment group.

### 3.5. Effects on *α*-SMA Expression

**Figure 5:**
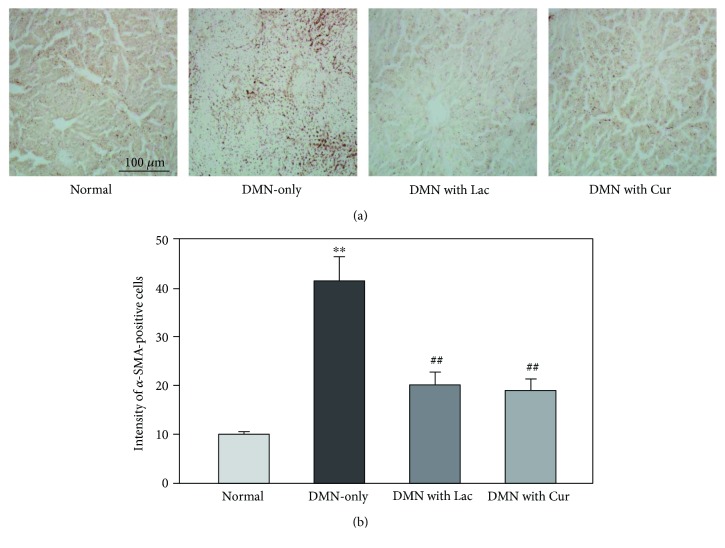
Effect of curcumin on *α*-SMA expression in liver tissue. (a) Representative photographs showing immunostained *α*-SMA in rat liver. (b) Positive cell count showing expression of *α*-SMA in rat liver tissue. Data are represented by mean ± SEM (*n* = 6 in each group). Statistical significances are compared between the normal and DMN-only groups (^∗∗^*p* < 0.01) or between the DMN-only and DMN with treatment groups (^##^*p* < 0.01).

To evaluate hepatic stellate cell (HSC) activation in the experimental groups, *α*-SMA expression was compared using immunohistochemical staining (Figure 5). All experimental groups showed increased *α*-SMA signals compared with the normal control (Figure 5(a)). The highest expression of *α*-SMA in the DMN-only group represents the highest extent of liver injury. Compared with the DMN-only group, the lactulose and curcumin treatment groups showed reduced *α*-SMA expression. The *α*-SMA expression level was numerically compared by counting the number of cells showing positive expression versus the total cell count (Figure 5(b)). The level of *α*-SMA expression was highest in the DMN-only group. Compared with the DMN-only group, the curcumin treatment group showed significantly lower *α*-SMA expression than the lactulose treatment group.

### 3.6. Effects on COX-2 Expression

**Figure 6:**
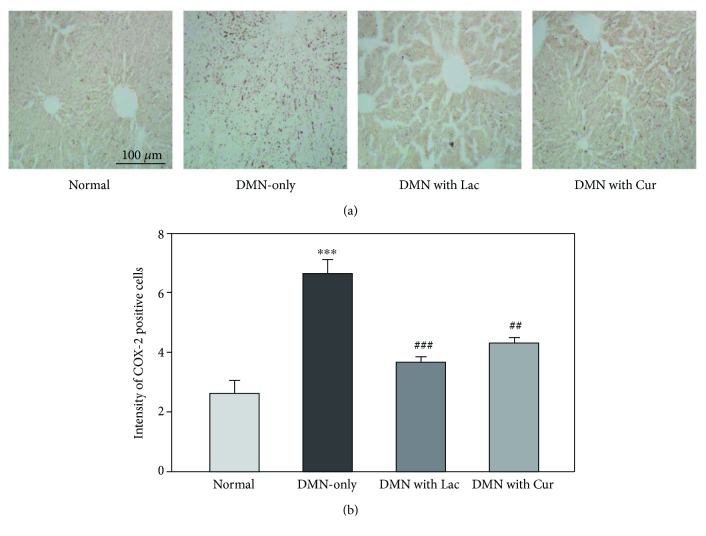
Effect of curcumin on COX-2 expression in liver tissue. (a) Representative photographs showing immunostained COX-2 in rat liver. (b) Positive cell count showing expression of COX-2 in rat liver tissue. Data are represented by mean ± SEM (*n* = 6 in each group). Statistical significances are compared between the normal and DMN-only groups (^∗∗∗^*p* < 0.001) or between the DMN-only and DMN with treatment groups (^##^*p* < 0.01; ^###^*p* < 0.001).

The degree of inflammation among the experimental groups was compared using the level of COX-2 expression (Figure 6). The DMN-only group showed the highest COX-2 expression, indicating a successful induction of the fibrotic liver model (Figure 6(a)). Curcumin and lactulose treatment of the fibrotic liver had a mitigating effect on liver inflammation, as demonstrated by the decreased COX-2 expression in Figure 6(a). The COX-2 expression level was numerically compared by counting the number of cells showing positive expression versus the total cell count (Figure 6(b)). The COX-2 expression level was highest in the DMN-only group. Compared with the DMN-only group, the lactulose and curcumin treatment groups showed significantly reduced COX-2 expression. The cell count in the DMN with curcumin group was slightly higher than that in the DMN with lactulose group, suggesting that curcumin also has a similar effect on the recovery of hepatic fibrosis as much as lactulose.

## 4. Discussion

In this study, we used DMN to induce liver cirrhosis to examine the ameliorating effect of herbal extracts on hepatic fibrosis. The general causes of chronic liver injuries and cirrhosis are alcohol, drugs, infections, autoimmune disease, vascular and metabolic disorders, biliary obstruction, and cryptogenic factors [17]. In rats, DMN induces broad micronodular cirrhosis with regenerative hepatocyte changes and bile duct proliferation. Phenomena such as increased mortality, destroyed hepatic parenchymal cells, and increased fibrotic tissue formation exhibited by DMN-treated rats are similar to the disease progression shown in human liver cirrhosis [18].

Histology of cirrhosis is typically characterized by diffuse fibrosis, regenerative nodules, and microvascular rearrangement [19]. The continuous hepatic damage can lead to fibrosis and cirrhosis, resulting in the hepatocytes eventually becoming surrounded by fibrous tissues. From a bioelectromagnetic perspective, this phenomenon is closely related to tissue conditions, including cellular structure, concentration and mobility of ions in intra- and extracellular structures, and other related factors [8–10]. At the initial stage of fibrosis, conductivity may increase owing to the excessive deposition of the extracellular matrix. Depending on the degree of fibrosis progression, conductivity will decrease owing to the damaged tissues, anisotropy, and liver stiffness of fibrosis [11, 12]. Electrical conductivity shows frequency-dependent spectra, so its values at different frequencies can provide different information. In this study, the MR-based electrical conductivity imaging method provided low-frequency conductivity information using externally injected currents. The advantage of low-frequency conductivity is that it provides information about cell membrane effects and tissue anisotropy according to the degree of cirrhosis. Several studies have reported that electrical conductivity provides unique information on tissue changes such as tumors, edema, ischemia, and liver fibrosis [9, 11, 12].

Our MREIT conductivity images of liver phantoms provide information about tissue condition by treatment effect. In the process of tissue damage by DMN injection, the movement and concentration of body fluids in the intra- and extracellular structure may influence the electrical conductivity. The lowest conductivity in the DMN-only group indicates relatively severe tissue damage caused by a substance toxic to the liver. The increased conductivities in the two treatment groups with lactulose or curcumin suggest a hepatoprotective effect on liver cirrhosis. The higher conductivity in the DMN with curcumin group compared with the lactulose treatment group indicates a more powerful anticirrhotic effect on the liver. In addition, the electrical tissue conductivity images correlated with the biochemical analysis results.

Chronic liver injuries cause apoptosis, and this results in compensatory regeneration of hepatocytes [20]. Injured hepatocytes release fibrogenic mediators and reactive oxygen species, induce HSC activation, and stimulate fibrogenesis [21]. In the normal liver, HSCs located between hepatocytes and sinusoidal endothelial cells are quiescent. However, in response to liver injuries, HSCs are transformed to active contractile myofibroblasts and contribute to microcirculation disturbances [22, 23]. Increases in HSC mobility and migration cause a process of sinusoidal remodeling [24], and excessive HSC function results in pericytic dysfunction, which is indicated by phenotypic markers such as *α*-SMA [25]. Curcumin induces apoptosis and suppresses proliferation in HSCs. In addition, it inhibits extracellular matrix formation by enhancing HSC matrix metalloproteinase expression via peroxisome proliferator-activated receptor gamma and by suppressing connective tissue growth factor expression [26]. Curcumin function in reducing HSC activation was confirmed by downregulation of *α*-SMA. COX-2 is expressed in macrophages and cancer cells and involved in situations such as acute and chronic inflammation, hemodynamics, tumorigenesis, renal function, and hepatic fibrogenesis [23, 27]. Whereas activated HSCs turn into myofibroblasts and express COX-2, isolated HSCs do not express COX-2 [28]. The hepatoprotective effect of curcumin on liver cirrhosis, which showed reduced hepatic inflammation and fibrogenesis, was supported by the finding that the increased COX-2 expression in the DMN-only treatment was significantly reduced by curcumin treatment.

In conclusion, the use of herbal medicine might be helpful in preventing liver fibrosis. Our results indicate that curcumin ameliorated liver cirrhosis via its anti-inflammatory effect and suppression of HSC activity, thereby attenuating fibrosis. Together with biochemical analysis, we used a MR-based electrical conductivity imaging method to enhance the detection of curcumin's hepatoprotective effect. Both the analysis of biochemical parameters and the comparison of electrical tissue conductivity showed that the effects of curcumin were comparable to those of lactulose. Future studies should address the mechanism of curcumin's antifibrotic effect and conduct *in vivo* imaging experiments to further investigate its clinical usefulness as a hepatoprotective treatment.

## Figures and Tables

**Table 1:** Measurement of body weight in four experimental groups.

Body weight (g)	Normal	DMN-only	DMN with Lac	DMN with Cur
Control	259.7 ± 2.0	269.4 ± 2.8	265.9 ± 2.5	270.4 ± 2.7
1 week	317.7 ± 7.0	320.0 ± 5.9	325.0 ± 4.9	330.1 ± 6.0
2 weeks	367.0 ± 6.2	360.9 ± 4.3	367.4 ± 1.8	365.3 ± 3.7
3 weeks	372.9 ± 6.7	350.9 ± 5.1	394.6 ± 9.1	370.9 ± 6.9
4 weeks	409.7 ± 8.4	373.9 ± 3.9	396.9 ± 8.7	383.6 ± 7.1

DMN: dimethylnitrosamine; Lac: lactulose; Cur: curcumin.

**Table 2:** Measurement of serum biochemical parameters in four liver tissues.

Parameter	Normal	DMN-only	DMN with Lac	DMN with Cur
AST (IU/L)	78.67 ± 15.69	128.33 ± 21.57^∗^	106.00 ± 37.24	72.33 ± 21.16^#^
ALT (IU/L)	42.33 ± 6.11	67.33 ± 8.73^∗^	45.00 ± 6.00^##^	38.67 ± 3.22^#^
ALB (g/dL)	3.93 ± 0.54	3.81 ± 0.77	3.70 ± 0.35	3.75 ± 0.63

Data are represented by mean ± SEM (*n* = 6 in each group). Statistical significances are compared between normal versus DMN-only (^∗^*p* < 0.05), DMN-only versus DMN with Lac and with Cur (^#^*p* < 0.05; ^##^*p* < 0.01, resp.).
